# Towards pixel-to-pixel deep nucleus detection in microscopy images

**DOI:** 10.1186/s12859-019-3037-5

**Published:** 2019-09-14

**Authors:** Fuyong Xing, Yuanpu Xie, Xiaoshuang Shi, Pingjun Chen, Zizhao Zhang, Lin Yang

**Affiliations:** 1 Department of Biostatistics and Informatics, and the Data Science to Patient Value initiative, University of Colorado Anschutz Medical Campus, 13001 E 17th Pl, Aurora, Colorado 80045, United States; 2 J. Crayton Pruitt Family Department of Biomedical Engineering, University of Florida, 1275 Center Drive, Gainesville, Florida 32611, United States; 3 Department of Computer and Information Science and Engineering, University of Florida, 432 Newell Drive, Gainesville, Florida 32611, United States

**Keywords:** Nucleus detection, Microscopy images, Deep neural networks

## Abstract

**Background:**

Nucleus is a fundamental task in microscopy image analysis and supports many other quantitative studies such as object counting, segmentation, tracking, etc. Deep neural networks are emerging as a powerful tool for biomedical image computing; in particular, convolutional neural networks have been widely applied to nucleus/cell detection in microscopy images. However, almost all models are tailored for specific datasets and their applicability to other microscopy image data remains unknown. Some existing studies casually learn and evaluate deep neural networks on multiple microscopy datasets, but there are still several critical, open questions to be addressed.

**Results:**

We analyze the applicability of deep models *specifically* for nucleus detection across a wide variety of microscopy image data. More specifically, we present a fully convolutional network-based regression model and extensively evaluate it on large-scale digital pathology and microscopy image datasets, which consist of 23 organs (or cancer diseases) and come from multiple institutions. We demonstrate that for a specific target dataset, training with images from the same types of organs might be usually necessary for nucleus detection. Although the images can be visually similar due to the same staining technique and imaging protocol, deep models learned with images from different organs might not deliver desirable results and would require model fine-tuning to be on a par with those trained with target data. We also observe that training with a mixture of target and other/non-target data does not always mean a higher accuracy of nucleus detection, and it might require proper data manipulation during model training to achieve good performance.

**Conclusions:**

We conduct a systematic case study on deep models for nucleus detection in a wide variety of microscopy images, aiming to address several important but previously understudied questions. We present and extensively evaluate an end-to-end, pixel-to-pixel fully convolutional regression network and report a few significant findings, some of which might have not been reported in previous studies. The model performance analysis and observations would be helpful to nucleus detection in microscopy images.

**Electronic supplementary material:**

The online version of this article (10.1186/s12859-019-3037-5) contains supplementary material, which is available to authorized users.

## Background

Nucleus/cell detection is usually a prerequisite for nuclear/cellular morphology computation in microscopy and digital pathology image analysis. It can enable quantitative information measurement to better understand the biological system or disease progression [1–3]. Manual assessment of object detection is labor intensive or even impossible due to the large amount of collected image data, which is rapidly increasing [4, 5], and thus many computerized methods have been developed for microscopy image computing [6–8]. In particular, machine learning techniques have been widely used to detect individual nuclei or cells in various microscopy images. Nevertheless, conventional learning methods heavily rely on appropriate data representations, which often require sophisticated expertise and domain knowledge, to achieve desired detection accuracies. In microscopy imaging, it is not unusual to generate images that exhibit significant appearance variation (e.g., staining, scale, etc.) in a single set of experiments such that designing appropriate data representations would be very difficult. Furthermore, it might be necessary to re-design image representations for each new dataset, and this is a non-trivial task. Therefore, most methods solve the detection problem only in a limited context or require substantial effort to adapt the models to new situations [9].

Recently, deep neural networks (DNNs) have powered many aspects in computer vision and attracted considerable attention in biomedical image computing [10]. Instead of relying on non-trivial image representation engineering, DNNs directly deal with raw image data and automatically learns the representations for different tasks. Compared with hand-crafted image features, learned representations require slight or no human intervention and can better capture intrinsic information for image description [11, 12]. DNNs have been applied to nucleus/cell detection in different types of microscopy images, leading to improved performance compared to other methods [13]. However, DNNs usually require a large number of training data, which might be often unavailable in the medical domain. In particular, supervised models like convolutional neural networks (CNNs), which are the most widely used for object detection in microscopy image analysis, need massive individual object annotation that is more expensive to obtain. Even though a sufficient number of annotated images are available on one specific dataset, it is currently common to annotate new target training images, i.e., label the locations of individual nuclei or cells, and re-train the models when applying them to other datasets.

It has been witnessed that CNNs can produce very powerful generic descriptors for visual recognition tasks [14, 15]. Feature representations extracted from CNNs, which are trained on large-scale image datasets such as ImageNet [16], are readily applicable to different tasks on a diverse set of datasets [17, 18]. ImageNet pre-trained CNNs are also fine-tuned or used as feature extractors on medical image datasets [19–21]. However, there is very limited literature covering deep model adaptation and evaluation for nucleus/cell detection on a wide range of microscopy image data. Although [22] learns a CNN architecture with multiple-organ tissue images, there are still several important, open questions to be answered. Another single CNN is trained with both magnetic resonance (MR) and computed tomography (CT) image data for multi-task image segmentation [23], but the conclusion might not be applicable to nucleus detection in microscopy images because of different imaging modalities and tasks. In addition, a large amount of previous work applies CNNs to object recognition with a sliding window strategy, which might not be computationally efficient for nucleus localization in high-dimensional pathology and microscopy images containing hundreds or thousands of nuclei or cells.

**Fig. 1:**
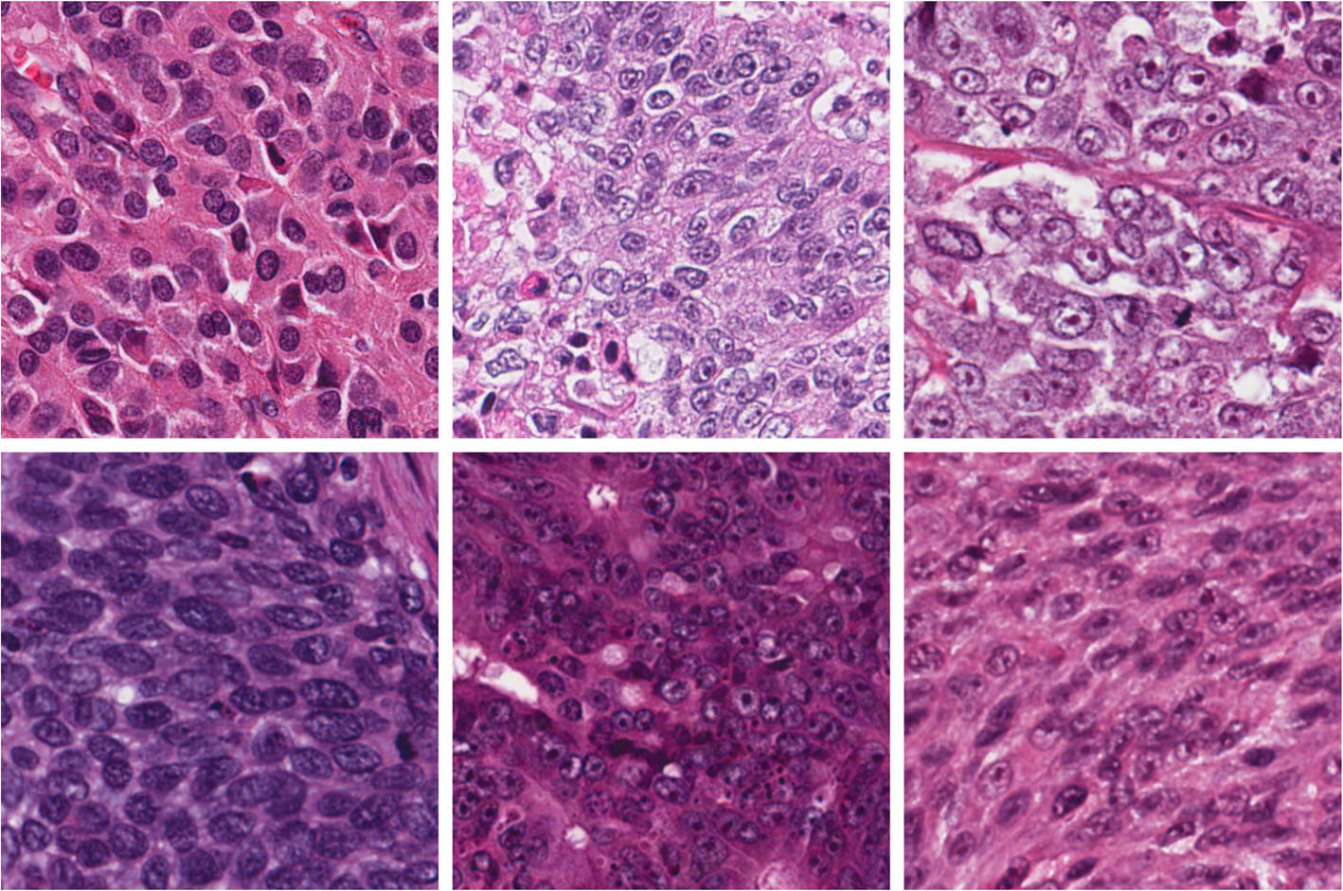
Network architecture. The black or red boxes denote feature maps, and the number of feature maps in each layer is also provided. The connections with different colors between feature maps represent distinct operations

**Fig. 2:**
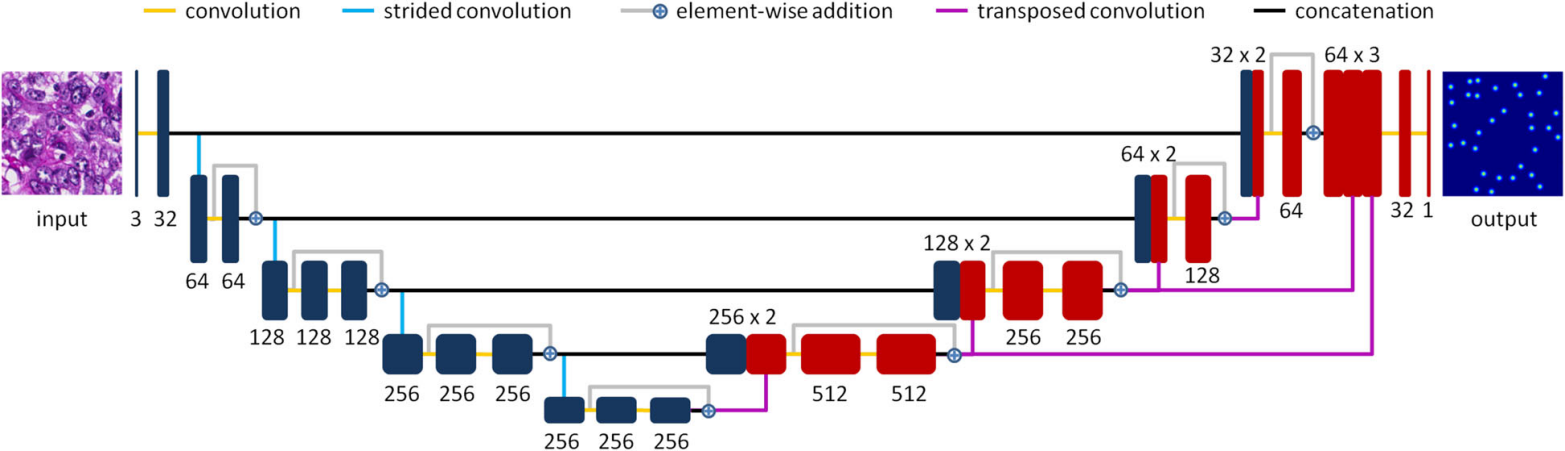
Sample images from different organs. Row 1 (from left to right): adrenal gland, bladder and breast; row 2: cervix, colorectum and eye. More details of data description can be found in the **Results** section

In this paper, we seek to answer two critical questions that have not been systematically studied yet: 1) Are deep nucleus detection models trained with one microscopy image dataset (i.e., images from one type of organ) applicable to other datasets (i.e., images from other organs), which are generated using the same staining technique and microscopy imaging protocol (see Fig. 2)? 2) For one specific organ dataset, will the use of image data from other organs for model training be helpful for a detection performance improvement? To this end, we present and extensively evaluate an end-to-end, pixel-to-pixel U-Net-like network (see Fig. 1) for nucleus detection in large-scale public pathology image datasets, The Cancer Genome Atlas (TCGA) [24]. In summary, the contributions are three-fold:

1) We observe that for a specific target dataset, training with images from the same types of organs might be usually necessary for nucleus detection. Although the images can be visually similar due to the same staining technique and imaging protocol, deep models learned with images from different organs might not deliver desired results and would require model fine-tuning to be on a par with those trained with target data.

2) We demonstrate that training with a mixture of target and other/non-target data does not always mean a higher accuracy of nucleus detection. A naive pooling of these two types of data might not be beneficial compared with target data alone, but learning with proper dataset balancing via loss function re-weighting could improve nucleus detection.

3) We conduct extensive experiments on 23 types of organ images from the publicly available TCGA pathology archive, which covers image data from different organs/cancer diseases and distinct institutions. We believe the findings from this systematic case study would be helpful to nucleus/cell detection in microscopy and digital pathology images.

### Related work

Deep networks have been successfully applied to medical image computing in different kinds of imaging modalities [25–27]. They have proven to be very effective in various image analysis tasks such as disease classification, lesion detection, object segmentation, image registration, tumor detection, etc [28–33]. DNNs also draw increasing attention in microscopy image analysis and a recent review can be found in [13]. Nucleus/cell detection, which is a critical step of image quantification in digital pathology and cell biology, is getting involved with deep learning and improved performance is emerging. Although different DNN architectures are used in medical image computing, CNNs and their variants are the dominant deep networks for object detection in microscopy images [13].

One straightforward method for nucleus/cell detection with DNNs is to conduct pixel-wise binary classification. Cireşan et al. [34] learn multiple CNNs with two types of small image patches (mitotic nuclei or not) and perform mitosis detection using a sliding window in hematoxylin and eosin (H&E) stained breast cancer images. Another CNN-based mitosis detection approach is presented in [35], and the difference is that it allows noisy data annotation by dealing with a data aggregation in the learning process. CNNs are also applied to nucleus/cell detection in other organ/tissue screening microscopy images such as brain, pancreas, bowel and circulatory systems [36–40]. Recently, a three-class CNN [22], which explicitly models nuclear boundaries, is introduced to detect nuclei in H&E stained images acquired from multiple organs. Stacked auto-encoder [41] is also applied to nucleus detection in breast cancer images, and it is first trained via unsupervised learning and then fine-tuned towards individual object detection. All of these approaches conduct pixel-wise prediction in a sliding window manner, which would be computationally expensive for high-dimensional images such as whole-slide scanned data.

In order to accelerate the algorithms, DNNs can be utilized to classify only nucleus/cell proposals instead of all image pixels. Dong et al. [42] apply CNNs to cell detection on region candidates, which are generated by a shallow model, support vector machine (SVM); Shkolyar et al. [43] first use simple image processing techniques to extract mitosis proposals and then exploit CNNs to conduct mitotic cell detection; Liu and Yang [44] assign CNN-predicted scores to cell candidates and then solves an inter linear programming problem for final cell detection in pancreatic neuroendocrine tumor (NET) and lung cancer images. Instead of relying on shallow models to extract regions of interest, Chen et al. [45] take advantage of fully convolutional networks (FCNs) followed by standard CNNs for mitosis detection. These methods avoid the expensive pixel-wise CNN predictions, but they require proper candidate collection, which might be usually challenging for histopathological images. Alternatively, a sparse kernel technique is incorporated into CNNs to reduce redundant computation [46, 47], and it has been applied to cell detection in lung cancer images.

Instead of performing independent pixel-wise classification, CNNs can take advantage of spatial topology to perform regression-based detection. Xie et al. [48] have replaced the classification layer with a structured regression in a conventional CNN such that the prediction can take into consideration adjacent information in the label space. This approach has been successfully applied to cell detection in multiple datasets including NET, breast cancer, and cervical cancer images. Another similar CNN-based spatial regression is presented in [49] for nucleus detection in colon cancer images and a CNN-based voting method is reported in [50], which learns an implicit codebook based on neighboring information for cell localization in NET pathology images. Regression modeling is also formulated with FCNs [51], which allow arbitrary-sized image inputs and enable efficient model inference, for fast cell detection in microscopy images [52, 53]. More recently, an FCN network with two sibling branches is proposed for simultaneous nucleus detection and classification [54] and the joint learning allows both tasks to benefit from each other. Another FCN-based cell detection method can be found in [55], where it introduces deconvolutional layers to the ResNet [56] such that the output probability map has an identical dimension as the input image.

## Results

### Implementation details

We implement the model with PyTorch [57] on a PC machine with a 3.50 GHz Intel i7 CPU and an Nvidia GeForce GTX 1080 Ti GPU. We train the model using stochastic gradient descent with Nesterov momentum and set the parameters as: learning rate=0.01, momentum=0.9, weight decay= 10^−6^, batch size=4 and number of iterations= 10^5^. The learning rate will decrease by a factor of 10 if the performance on the validation sets does not improve for 10^4^ iterations until it is smallar than 10^−4^. We set *α*=3, *d*=15 in Eq. (1) and *λ*=5 in Eq. (2). Following [52, 53], we scale the proximity values by a factor (i.e., 5) to facilitate training. The hyperparameter of the exponential linear unit (ELU) is set as 1. Dropout [58] with a rate of 0.5 is used after the convolution operations in the last two residual blocks of the downsampling path.

For model training, we randomly crop four 200×200×3 image patches from each training image to form the training sets. We normalized the patches by stracting mean and dividing standard deviation in each image channel. We adopt data augmentation including random rotation, shifting, mirroring and elastic distortion to prevent overfitting. In order to save storage space, we dynamically crop image patches within each iteration.

### Datasets

We collect 23 types of H&E stained tissue image data from the public TCGA Research Network [24], with each containing 50 images and in total 1128 images (only 35, 44 and 49 images available for bile duct, lymph nodes and stomach respectively), one per patient. Each category corresponds to one specific organ/cancer disease, and it covers image data from multiple institutions. Thus, in total we have 23 different image datasets. For one patient of each dataset, only one 500×500×3 image patch (in this paper, we simply use images for description) is cropped from the whole-slide image, which is generated with digital microscopy imaging at 40x magnification. A few example images are displayed in Fig. 2, which exhibit significant challenges including background clutter, inhomogeneous intensity, nucleus touching/overlapping, scale variation, etc. For each dataset, we randomly split the image data into two halves, one for training and the other for testing. We further randomly select 20% of training data (i.e., 5 images) as a validation set. There is no overlapping between any two sets of training, validation and testing. For all the images, the gold-standard nucleus centers are manually annotated.

### Evaluation metrics

We use the evaluation metrics [52] in the experiments. Specifically, we define the circular region with a 16-pixel radius centered at each annotated nucleus centroid as its gold-standard region. For a test image, the detected points within the gold-standard regions are matched with corresponding annotated nucleus centroids by using the Hungarian algorithm [59], which is to find an assignment of detections to annotations with a minimal cost. The cost of a detection to a human (or gold-standard) annotation is defined as the Euclidean distance between these two points. After the assignment, the detections matched with human annotations are considered true positives (TP), and those detections not matched with any annotations are false positives (FP). The human annotations that do not have matched detections are viewed as false negatives (FN). Based on these definitions, we report detection accuracy using precision (P), recall (R) and *F*_1_ score: $P=\frac {TP}{TP+FP},\ R=\frac {TP}{TP+FN},\ F_{1}=\frac {2\times P\times R}{P + R}$.

### Nucleus detection evaluation

#### Baseline experiments

**Fig. 3:**
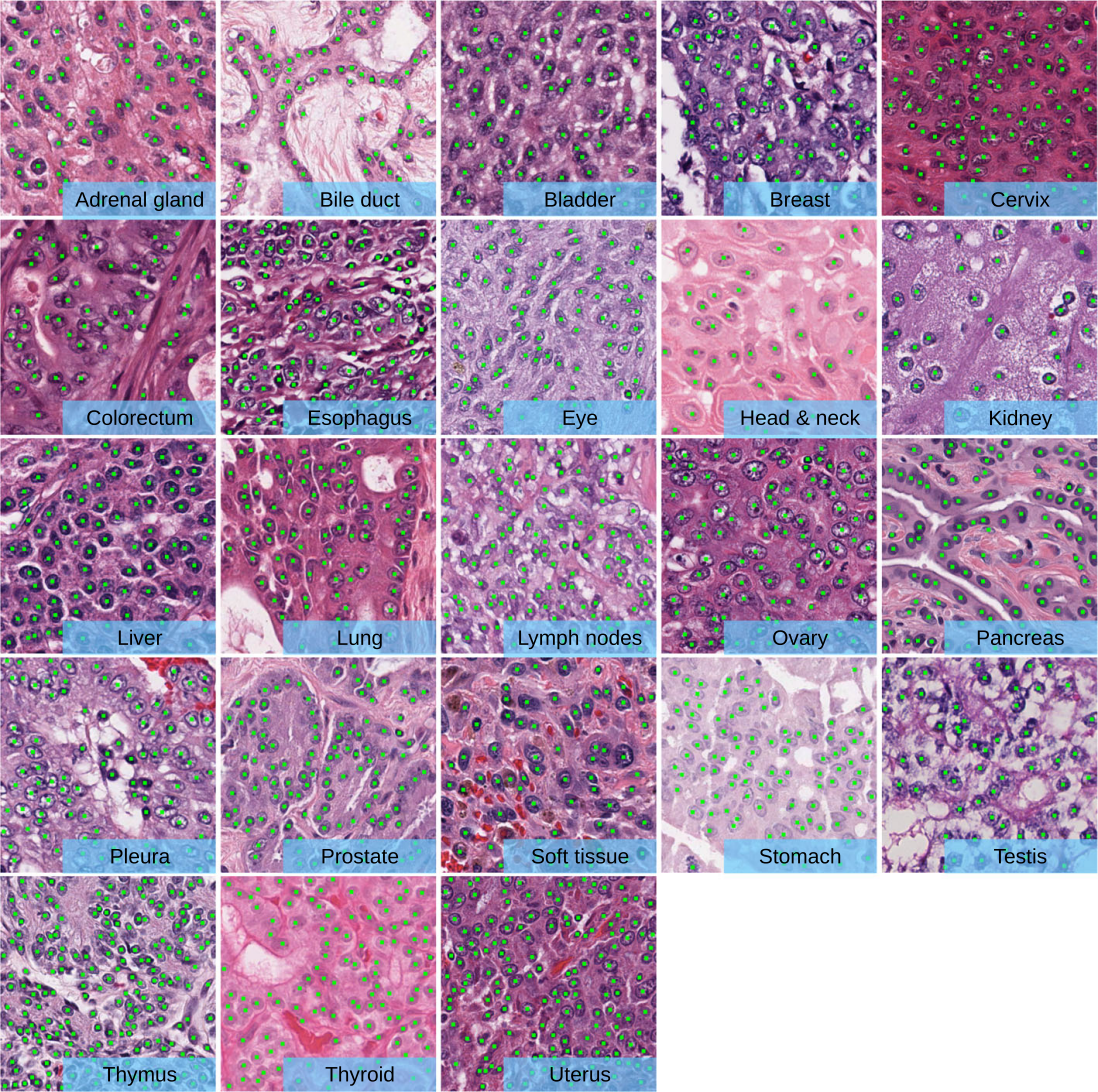
Qualitative results of MicroNet. Nucleus detection is marked with green dots on example images. These images exhibit the difficulty of nucleus localization due to significant challenges, such as background clutter, inhomogeneous intensity, object touching/overlapping, dense object clustering, scale and shape variations of objects, etc

To set up a baseline, we train the proposed FCN regression model, referred to as MicroNet, on all the 23 datasets and compare it with other recent state-of-the-art deep methods such as FRCN [52], FCRNA [53], FCRNB [53], U-Net [60] and FCN [51]. Here we select these pixel-to-pixel learning and inference models for a fair comparison. We evaluate each method on the testing sets, where the optimal value for *ξ* is determined by calculating the best *F*_1_ score on the validation set in each dataset. Additionally, we measure the detection using the Euclidean distance (ED) between TP and matched gold-standard annotations. Table 1 shows the mean and standard deviation of each metric for different methods over all the 23 datasets. As we can see, MicroNet produces the highest *F*_1_ score and the lowest ED. In particular, MicroNet outperforms FCRNB, U-Net and FCN by a large margin in terms of the *F*_1_ score, which is a unary measurement for object detection. Interestingly, the pixel-wise classification models, U-Net and FCN, produce significantly lower recall compared with the regression models, probably due to a high FN rate. FCRNA and FCRNB exhibit better recall but lower precision, and the recent deep regression model FRCN provides a good tradeoff between precision and recall. MicroNet provides a slightly better *F*_1_ score than FRCN but a much lower ED, which means MicroNet can deliver more accurate nucleus localization. These observations demonstrate that MicroNet is readily suitable for further study. Figure 3 shows qualitative results of nucleus detection using MicroNet on several example images.

**Table 1:** Nucleus detection (mean ± standard deviation) over the 23 datasets in terms of precision, recall, *F*_1_ score, and Euclidean distance

	Precision (%)	Recall (%)	*F*_1_ score (%)	Euclidean Distance
FCN	79.7 ±6.4	74.0 ±8.2	76.4 ±5.7	5.05 ±0.92
U-Net	83.0 ±6.6	71.7 ±7.0	76.6 ±4.5	6.01 ±0.72
FCRNA	73.7 ±8.3	86.6 ±6.8	79.3 ±5.4	4.12 ±0.84
FCRNB	68.6 ±9.4	87.5 ±6.8	76.5 ±6.9	4.36 ±1.19
FRCN	75.4 ±6.4	88.2 ±3.8	81.2 ±4.5	5.17 ±0.52
MicroNet	76.7 ±7.5	87.8 ±4.6	81.6 ±4.9	3.49 ±1.04

#### Generalization on different datasets

**Fig. 4:**
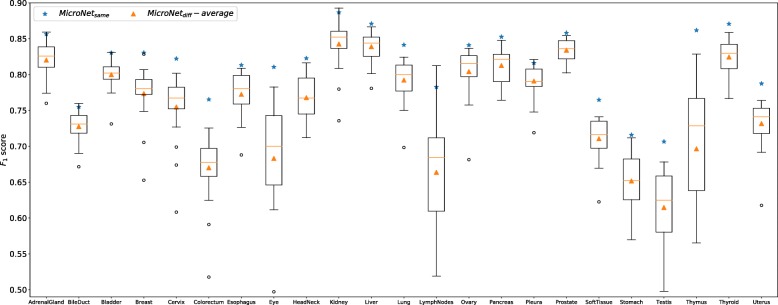
The *F*_1_ score of MicroNet on different data. Blue stars denote models trained and tested on the same datasets (*MicroNet*_*same*_), i.e., images from the same types of organs across different patients. The boxes represent models trained on one dataset but tested on another and the orange triangles denote the average performance (*MicroNet*_*diff*_-*average*) over these models

For each dataset, we train one individual MicroNet model and apply it to the testing sets from both the same and other datasets. In other words, we test MicroNet using images from not only the same types of organs but also distinct ones, which are not used for model training. Figure 4 shows the *F*_1_ score of MicroNet on each individual dataset. For most datasets, models trained and tested on the same categories of organs, denoted by *MicroNet*_*same*_, produce better detection accuracies than those trained and tested on different organ images, denoted by *MicroNet*_*diff*_. Interestingly, for adrenal gland, bile duct, kidney, lymph nodes, and pleura datasets, there are models trained on certain other organ images providing slightly higher *F*_1_ scores than *MicroNet*_*same*_; however, *MicroNet*_*same*_ produces competitive performance to the best models on these datasets. We also observe that for each individual dataset, the *F*_1_ score of *MicroNet*_*same*_ is much higher than the average *F*_1_ score of *MicroNet*_*diff*_ across all the other datasets, and many *MicroNet*_*diff*_ models provide much lower accuracies than *MicroNet*_*same*_. This suggests for one specific dataset, learning with other organ images is not necessary to deliver desired nucleus detection results, although all the tissue images are generated with H&E staining and digital microscopy. The precision-recall curves of MicroNet models on all 23 datasets are provided in Additional file 1: Figure S1.

**Fig. 5:**
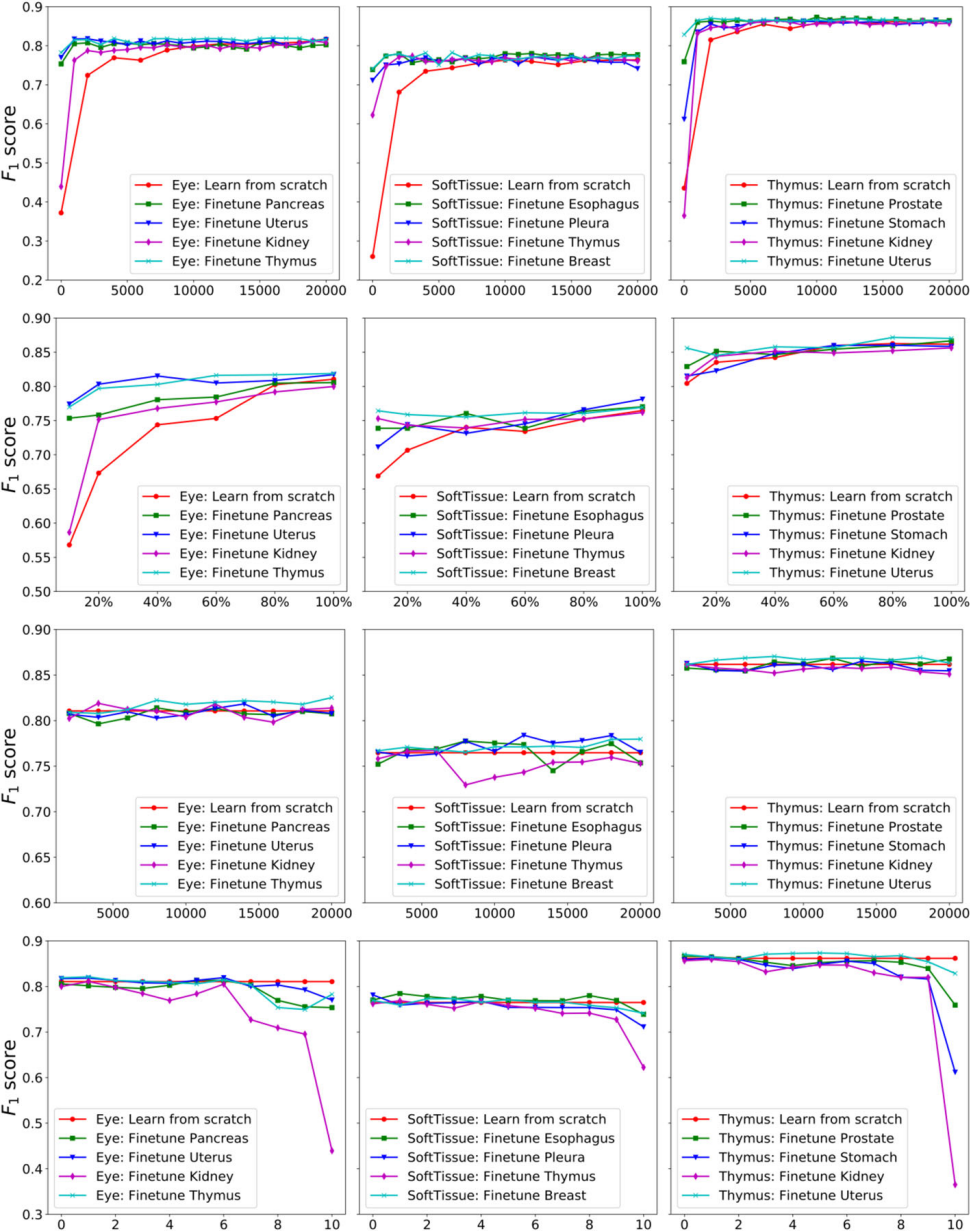
Performance of MicroNet fine-tuning. From top to bottom, each row represents the *F*_1_ score of model fine-tuning with respect to the number of training iterations (row 1), the percentage of target training data (row 2), the stage of base models (row 3) and the number of fixed learning blocks during fine-tuning (row 4). For a comparison, the *F*_1_ score of learning from scratch is also provided in each plot

We further explore whether training on one dataset can be beneficial to nucleus detection on other datasets via model fine-tuning. To this end, we compare MicroNet fine-tuning to learning from scratch on different datasets. Due to the large number of combinations from the entire set of all data, we choose a subset of data to conduct experiments. Specifically, we randomly select 3 target datasets and 6 base datasets, each two corresponding to one target dataset. Based on Fig. 4, we also choose the non-target datasets that produce the highest and lowest *F*_1_ scores for each target dataset as two additional base training sets, as shown in Table 2. For simplifying descriptions, these two types of datasets are called the best and worst base datasets for each target data respectively. We train one MicroNet model on each base dataset and then fine-tune it towards the corresponding target data. Here we fine-tune the entire neural network instead of freezing some layers. We compare these models to those directly learned from scratch on target data in the first row of Fig. 5. We note that model fine-tuning can perform as good as learning from scratch with much less training time, no matter from which base dataset it conducts the fine-tuning. It means model fine-tuning might have a lower requirement of the number of iterations for training convergence.

**Table 2:** Base datasets for model fine-tuning

Target data	Base data (random)	Base data (worst)	Base data (best)
Eye	Pancreas, Uterus	Kidney	Thymus
Soft tissue	Esophagus, Pleura	Thymus	Breast
Thymus	Prostate, Stomach	Kidney	Uterus

The second row of Fig. 5 compares model fine-tuning to learning from scratch on different numbers (i.e., 10*%*, 20*%*, 40*%*, 60*%*, 80*%* and 100%) of target training data. With the increasing of target training data, both of these two learning strategies improve the nucleus detection accuracies, and this suggests learning with more target data is beneficial. More important, fine-tuning can achieve a desired detection accuracy with less target training data than learning from scratch. In particular, only 20% of eye target training images to obtain a 0.80 *F*_1_ scorefor fine-tuning from the uterus base dataset, while learning from scratch needs 80% of the eye training images. These experimental results demonstrate fine-tuning MicroNet could achieve a specific nucleus detection accuracy with limited training data. Therefore, it can reduce human effort for training data annotation and enable high-throughput image quantification when applying MicroNet to different datasets. We also explore when it is ready for MicroNet model fine-tuning. If early transfer can provide competitive performance to fine-tuning from the optimal base models, it might significantly reduce the computational cost of training on base datasets. This would be particularly helpful when base datasets are large. Specifically, we take a snapshot of models trained on base datasets at every 2000 iterations and then fine-tune these base models towards corresponding target datasets, as shown in the third row of Fig. 5. We find that fine-tuning early-stage base models (e.g., no more than 6000 iterations) can provide similar performance to those learned from scratch. For most cases, early transfer is competitive to late transfer. Meanwhile, fine-tuning late-stage models from the best base datasets always outperforms learning from scratch in the selected datasets.

In order to evaluate the transferability of network layers on nucleus detection, we freeze the first several learning blocks during model fine-tuning. Figure 1 shows that in addition to the downsampling and upsampling paths, each of which has four residual learning blocks, MicroNet has one input (one convolutional operation) and one output (two convolutional operations) transition blocks. From input to output, we label all the blocks from 1 to 10. The fourth row of Fig. 5 demonstrates the *F*_1_ scores of model fine-tuning with keeping different blocks frozen. For most cases (except fine-tuning from esophagus towards soft tissue), fine-tuning only the last 2 or 3 blocks provides lower accuracies than learning from scratch. On the other hand, fine-tuning can improve the performance with less blocks fixed. When only the first 2 blocks are frozen, fine-tuning can compete with or even outperform learning from scratch.

#### Training with auxiliary datasets

**Fig. 6:**
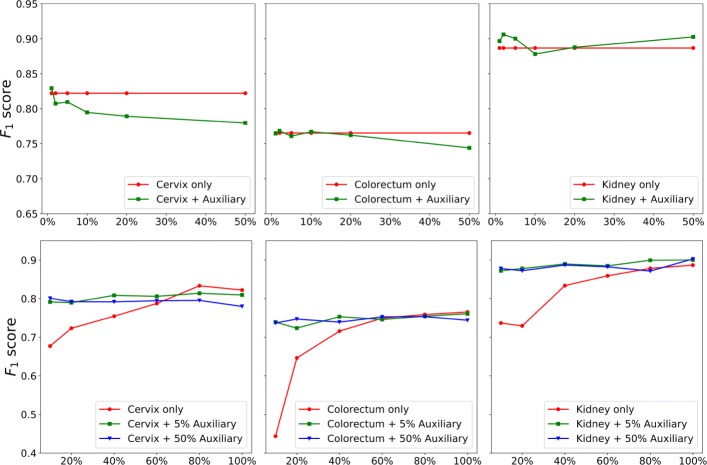
Comparison between MicroNet learning with and without auxiliary data. For each target dateset (i.e., cervix, colorectum or kidney) in the top row, all its training data are mixed with different numbers of auxiliary training data (*x*-axis represents the percentage of auxiliary training data). In the bottom row, a fixed number (i.e., 5% and 50%) of auxiliary training data are used to mix with different numbers of target training data, as shown in the *x*-axis

In this experiment, we evaluate whether training with a mixture of different datasets is beneficial. To this end, we randomly choose 3 target datasets, i.e., cervix, colorectum and kidney, and mix each training data with corresponding auxiliary data. For one target dataset such as cervix, all the other 22 non-cervix datasets are pooled to form the auxiliary training data, from which we randomly select 1*%*, 2*%*, 5*%*, 10*%*, 20*%* and 50% to mix with the target training data respectively. We train one MicroNet for each of these mixed training data (denoted by *MicroNet*_*mix*_) and compare it to the one learned with only the target training set (denoted by *MicroNet*_*target*_), as shown in the top row of Fig. 6. For cervix and colorectum datasets, the *F*_1_ score of *MicroNet*_*mix*_ decreases as the increasing of auxiliary training data and becomes lower than that of *MicroNet*_*target*_; for kidney, the score of *MicroNet*_*mix*_ first decreases and then increases to be higher than that of the counterparts. These observations suggest that a mixture of target and non-target datasets might not be always helpful for nucleus detection on one specific target dataset, even though all the images are generated with the same microscopy imaging protocol and H&E staining technique.

In order to explore whether training with auxiliary data is helpful when target data are limited, we train multiple models with different numbers of target training images. For each of the aforementioned 3 target datasets, we randomly generate multiple training sets with 10*%*, 20*%*, 40*%*, 60*%*, 80*%* and 100% of the original training data respectively; meanwhile, we randomly select 5% and 50% of auxiliary training data and mix each with the generated target training data to form new training sets. The bottom row of Fig. 6 shows a comparison between training with and without auxiliary data. Clearly, for the 10*%*, 20*%* and 40% target training sets, training with only target data produces poor performance probably due to overfitting, while learning with a mixture of target and auxiliary data provides significantly better results. However, learning with too much auxiliary data (i.e., 50%) might overwhelm the target data such that the detection accuracy would decrease, as illustrated in the plot for the cervix dataset in Fig. 6.

**Fig. 7:**
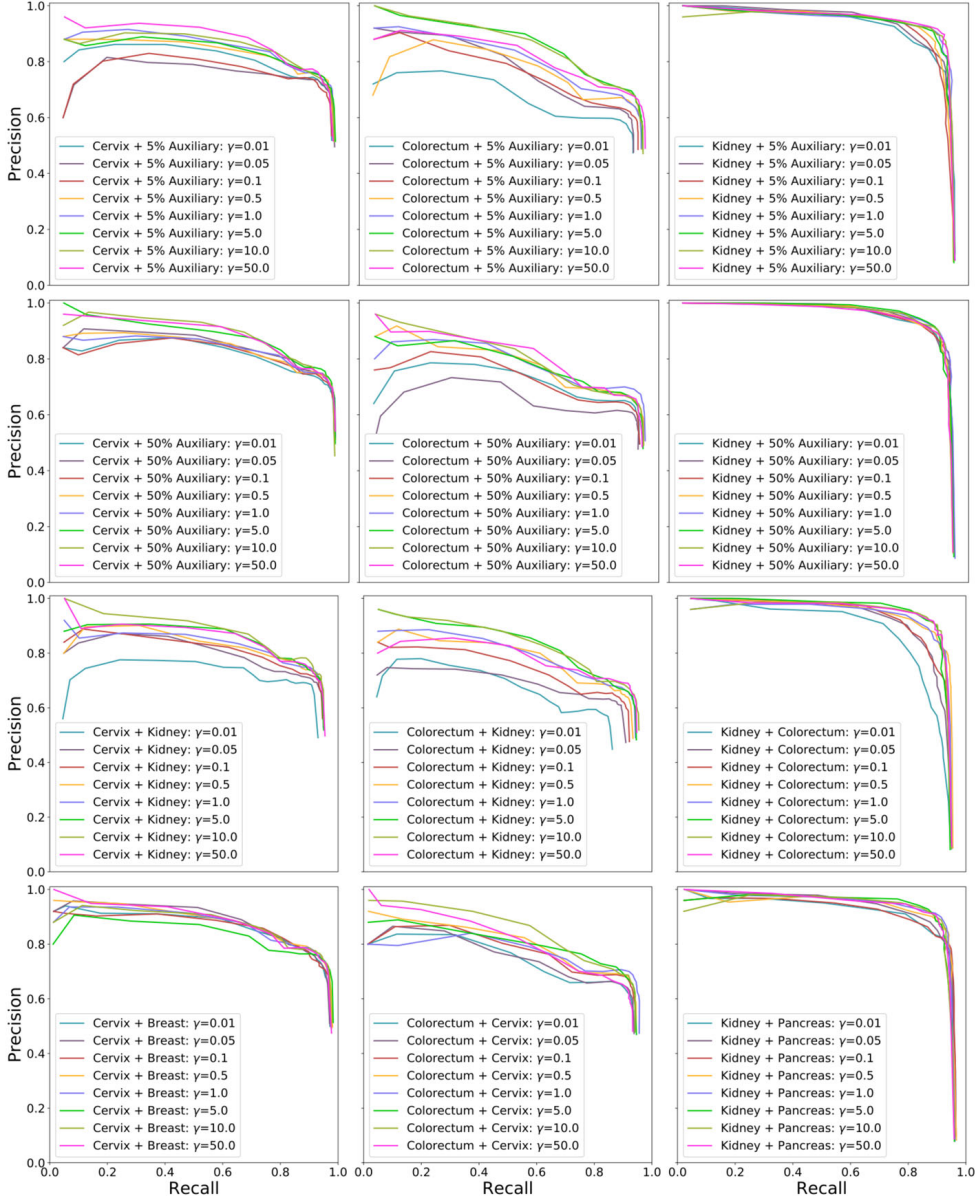
Precision-recall curves with different *γ* values on different training data. The top two rows represent 5% (row 1) and 50% (row 2) of mixed auxiliary data respectively, and the bottom two rows denote two different single-source auxiliary data respectively, which are the base datasets producing the lowest (row 3) and highest (row 4) *F*_1_ score on the target data in Fig. 4. Each curve is generated by varying the threshold *ξ*. *x*/*y*-axis represents recall/precision. For each curve label of “A+B", A and B represent target and auxiliary data respectively

**Fig. 8:**
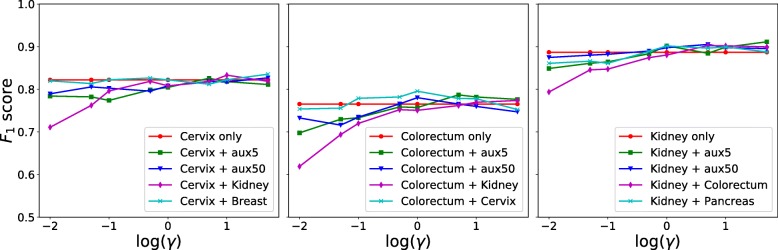
The *F*_1_ score with different *γ* values on different training data. *x*/*y*-axis represents *F*_1_ score/ log(*γ*). For each curve label of “A+B", A and B represent target and auxiliary data respectively

In the previous experiments above, the auxiliary data can have a much larger size than the target data and dataset balancing during model training might be helpful for performance improvement. Thus, we further evaluate nucleus detection based on dataset balancing with weighting the loss. Specifically, we minimize the weighted sum of two losses, $\mathcal {L}=\gamma \mathcal {L_{T}} + \mathcal {L_{A}}$, where $\mathcal {L_{T}}$ and $\mathcal {L_{A}}$ are target and auxiliary losses respectively, and both of them use the definition in Eq. (2). *γ* is a control parameter balancing the contributions from the two data sources. For each target dataset, we use all of its training images as the target training set and randomly select 5% and 50% of auxiliary training data as the auxiliary training set respectively. The top two rows of Fig. 7 show the precision-recall curves with respect to different *γ* values. We can see that for either 5% or 50% of auxiliary data, a small *γ* value (e.g., less than 1.0) leads to poor performance, especially for cervix and colorectum datasets, perhaps because model training mainly relies on the auxiliary data. The performance improves with the increasing of *γ* values. In addition, compared to the naive pooling (i.e., *γ*=1.0) of target and auxiliary training sets, learning with larger weights on target data (i.e., *γ*>1.0) can produce better nucleus detection. We also find that there is no significant performance variation for the kidney dataset with 50% of auxiliary data. This observation is consistent with those in Fig. 6, where models learned with auxiliary data is helpful for nucleus detection in kidney data and actually slightly outperforms the models trained with only target data. Figure 8 shows the *F*_1_ scores with different *γ* values. As expected, learning with more emphasis on auxiliary data, i.e., *γ*<1.0, leads to lower detection accuracies than those trained with naive data pooling or only target data (denoted by *MicroNet*_*target*_). However, learning with a proper weighting of target data might improve the performance and be on a par with or even outperform *MicroNet*_*target*_.

We also evaluate learning with a single auxiliary data source and compare it to those with the mixed multi-source auxiliary data above. For each target data, i.e., cervix, colorectum and kidney, we select the base datasets that producing the lowest and highest *F*_1_ scores in Fig. 4 as two single-source auxiliary data: kidney/breast for cervix, kidney/cervix for colorectum, and colorectum/pancreas for kidney. The bottom two rows of Fig. 7 show the precision-recall curves with different *γ* values on these single-source auxiliary data. Similar to learning with multi-source auxiliary data, it exhibits poor performance when *γ*<1.0 and the detection accuracy improves as the increasing of *γ* values. Meanwhile, training with higher weights on target data (*γ*>5.0) leads to better performance. We also observe for single-source auxiliary data, learning with a proper *γ* value (e.g., larger than 1.0) might outperform those models learned with only target training data.

### Effects of parameters

**Fig. 9:**
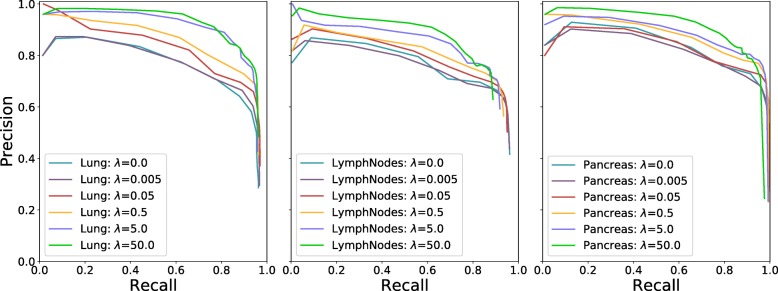
Precision-recall curves with different *λ* values. Each curve is generated by varying the threshold *ξ*. *x*/*y*-axis represents recall/precision

The parameter *λ* in Eq. (2) plays an important role on nucleus localization. We randomly select 3 datasets, i.e., lung, lymph nodes and pancreas, to evaluate its effects. Figure 9 shows the precision-recall curves with different *λ* values: *λ*=0, 0.005, 0.05, 0.5, 5 and 50. We do not include the performance for *λ*=500 and *λ*=5000 due to the exploding gradient problem. As we can see, the models with *λ*≤0.5 are outperformed by those with *λ*≥5.0; in particular, the model with *λ*=0, which indicates no additional penalty on the regions with nonzero values in the proximity maps, exhibits significantly worse performance than those with large *λ* values. This might be because for a single training image, a dominant portion of its proximity map has zero values and a penalty on the central regions of nuclei would enforce model learning to pay more attention to these nonzero-value regions. In this scenario, model inference would be encouraged to avoid trivial solutions and predict nonzero values on the central regions of nuclei.

### Evaluation of other deep models

**Fig. 10:**
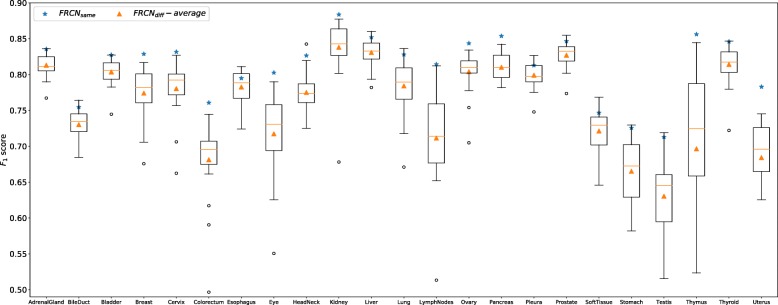
The *F*_1_ score of FRCN on different data. Blue stars denote models trained and tested on the same datasets (*FRCN*_*same*_), i.e., images from the same types of organs across different patients. The boxes represent models trained on one dataset but tested on another and the orange triangles denote the average performance (*FRCN*_*diff*_-*average*) over these models

**Fig. 11:**
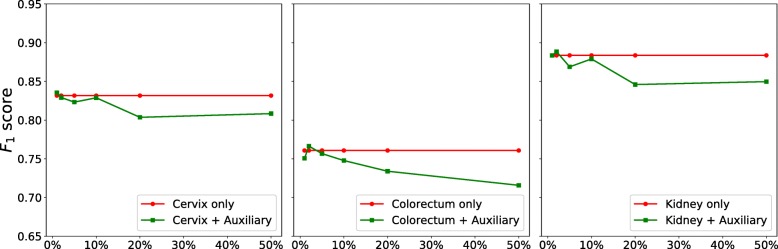
Comparison between FRCN learning with and without auxiliary data. For each target dateset (i.e., cervix, colorectum or kidney), all its training data are mixed with different numbers of auxiliary training data (*x*-axis represents the percentage of auxiliary training data)

We further explore whethter other deep nucleus detection models exhibit similar performance. Here we choose a very recent state-of-the-art model, FRCN [52], to evaluate its generalization on different datasets. Specifically, we train one FRCN model for each type of organ data and apply it to nucleus detection on the same- and different-organ images. Figure 10 shows the *F*_1_ score of FRCN on all the datasets. Similar to MicroNet, *FRCN*_*same*_ produces better performance than many *FRCN*_*diff*_ on each dataset, and its *F*_1_ score is significantly higher than the average *F*_1_ score of *FRCN*_*diff*_. This observation is consistent with that for MicroNet, i.e. training models with a specific dataset might not provide desired results on other datasets and learning with the same type of organ might be usually preferred. We also evaluate whether learning FRCN models with auxiliary datasets is helpful for nucleus detection. Following the experimental setting in Fig. 6 (the top row), we compare the models trained with target data only to those learned with mixed datasets, which contain all target training data and different numbers of auxiliary images. Figure 11 shows that learning FRCN models with a mixture of target and non-target data might not always improve nucleus detection, and this is also consistent with the study of MicroNet above.

## Discussion

On the basis of Figs. 4 and 5, we observe that models learned on one type of organ images might perform poorly in other organ datasets; however, model fine-tuning from other organ data can provide similar performance to those directly trained with target data from scratch in a relatively shorter period of time. In addition, fine-tuning models from the best base datasets requires slightly smaller iteration numbers than learning from the worst base datasets, e.g., 2000 versus 5000. For the target datasets, fine-tuning usually achieves stable performance within only 5000 iterations, while training from scratch needs over 15000 iterations. These observations show model fine-tuning is more efficient when base models trained with other datasets are available, and this condition might be usually satisfied in real applications. We also find that compared with learning from scratch and fine-tuning from the worst base datasets, fine-tuning from the best base datasets provide slightly better detection accuracies, especially when insufficient target training data are available. This suggests a proper selection of base datasets might be important for model fine-tuning from limited target data. Interestingly, the *F*_1_ score might decrease when fine-tuning late-stage models from the worst base datasets, as shown in soft tissue and thymus subplots (row 3 of Fig. 5). This might be because these base models are well trained and too specific to the base datasets.

From Figs. 6, 7 and 8, we find that learning with mixed target and auxiliary data might be not always beneficial. However, using a certain amount of auxiliary data to assist model training for nucleus detection might be helpful when only a small target training set is available. We also observe that for either multi-source or single-source auxiliary data, it is critical to balance the datasets during model training. Interestingly, learning with the best auxiliary data (row 4 of Fig. 7) provides better nucleus detection than the other single-source auxiliary learning (row 3 of Fig. 7), especially when *γ*<1.0, as shown in Fig. 8. This suggests it might be critical to choose auxiliary dataz when using a single auxiliary source, which has a similar size to the target set.

## Conclusions

In this paper, we address several important but previously understudied questions on deep models for nucleus detection in microscopy images. We present and evaluate an end-to-end, pixel-to-pixel FCN model for nucleus detection on a wide variety of digital pathology image data, which cover 23 types of different organs/diseases. All images are H&E stained and digital microscopy at 40× magnification. The datasets are collected from multiple institutions and should be sufficiently diverse. We find that for a specific target dataset, i.e., images from one type of organ, training with images from different organs might not deliver desired results, even although the images are generated using the same staining technique and imaging protocol. Our experiments further demonstrate model fine-tuning or transfer learning is more efficient compared to training from scratch. To achieve a desired object detection accuracy, model fine-tuning requires less target training data or a smaller number of training iterations.

We also observe learning with auxiliary data might be helpful for nucleus detection, but it does not always mean higher accuracies. When there are limited target training data, a naive mixture of target and auxiliary data would be helpful since it could address the overfitting problem; however, this naive data pooling might not be always beneficial if sufficient target training data are available. On the other hand, learning with dataset balancing can provide better nucleus detection than training with a simple pooling of target and auxiliary data. With an appropriate data weighting, it would be able to provide competitive or even higher detection accuracies than training with only target data. We also show learning with more emphasis on the central regions of nuclei is helpful for nucleus detection in microscopy images.

## Methods

### Network architecture

Our model is shown in Fig. 1, which can be viewed as a variant of FCNs. It is mainly inspired by the residual regression network [56] and U-Net [60], and the major difference is that we aggregate different levels of contextual information for robust pixel-wise prediction. The network consists of four basic paths: downsampling, upsampling, concatenation and multi-context aggregation. The downsampling path aims at extracting hierarchical features from input images, while the upsampling path maps feature representations into the input space for dense prediction. In order to preserve the high-resolution information for object localization, low-layer feature maps from the downsampling path are copied and concatenated with corresponding representations in the upsampling path. Finally, a multi-context aggregation is introduced to ensemble contextual information such that the model can handle scale variation of nuclei.

The downsampling path consists of a stack of residual learning blocks [56], which learn feature representations via a residual mapping instead of the original underlying mapping such that gradients would not vanish in backpropagation. Specifically, a shortcut connection is used to realize an identity mapping, which is added to a non-linear mapping (i.e., convolution followed by an activation) for residual learning in an element-wise way. A strided convolution with a stride of 2 is exploited to downsample feature maps between adjacent residual blocks. Batch normalization [61] is applied after each convolution and strided convolution. Subsequently, an ELU [62] is used for the non-linear transform after each batch normalization and element-wise addition. After the input transition block that consists of one convolutional layer, four residual blocks are stacked to learn high level abstraction information by following the rules [63]: 1) double the number of convolutional filters when the feature map size is halved (except for the last residual block) and 2) the filter size is set as 3×3 with the padding being 1 in each residual block. The upsampling path is also built with four cascaded residual blocks, but in a converse direction of the downsampling configuration. A transposed convolution [64] instead of a strided convolution is applied to the connection of residual blocks, aiming at increasing the resolution of learned high-level feature maps for pixel-wise prediction.

In order to compensate for high-resolution information loss due to strided convolutions, we use concatenation connections [60] to combine feature maps from both downsampling and upsampling paths. Specifically, downsampled outputs are copied and linked to corresponding upsampled outputs such that a successive layer can learn to fuse this information for precise object localization. It is worth noting that these feature maps might not be directly concatenated with each other, because the downsampling and upsampling layers could be not exactly symmetric. For instance, a 75×75 feature map after downsampling with a factor of 2 would have a dimension of 37×37 (without loss of generality, the floor operation is used); however, a 37×37 feature map after upsampling would have a size of 74×74. In order to preserve a proper output size, we pad upsampled outputs with zeros to match downsampled ones for information fusion.

Due to object scale variation, network prediction based on a single-sized receptive field might not well localize all the nuclei. Inspired by [65], we introduce a multi-context aggregation path to assemble different levels of feature maps for final pixel-wise prediction. Since downsampled outputs in different layers correspond to distinct-sized receptive fields and those in deeper layers have larger receptive fields, we can take advantage of this contextual information by combining the hierarchical feature representations. More specifically, we directly apply transposed convolutions to the downsampled outputs at certain layers (see Fig. 1) and aggregate the generated feature representations to form a multi-context feature map, which is fed to the output transition block (consisting of two convolutional layers) for final output prediction.

### Model formulation

**Fig. 12:**
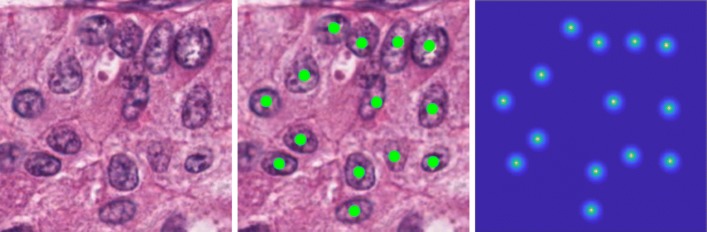
Proximity map generation. From left to right: the original image, manual annotation (green dots) of nucleus positions and proximity map, where the central regions (light blue) of nuclei have continuous, nonzero values and all the other regions are assigned zero values (dark blue)

In this paper, nucleus detection is formulated as a regression problem. Compared to binary classification, regression modeling can employ additional context during the learning stage for more accurate detection [52, 66]. Our goal is to learn an FCN regressor to predict an identical-sized proximity map given an input image, where each predicted pixel value measures how proximal this pixel is to its closet nucleus center. To this end, we define gold-standard proximity maps (or structured labels) based on the Euclidean distance. For a *w*×*h* training image $\mathbf {x}^{i}\in \mathbb {R}^{w\times h\times c}$ with human annotation of nucleus centers, where *c* is the number of image channels, we generate its corresponding proximity map $\mathbf {y}^{i}\in \mathbb {R}^{w\times h}$ as follows

1 $$\begin{array}{@{}rcl@{}} y^{i}_{uv} = \left\{\begin{array}{ll} \frac{e^{\alpha(1-\frac{D^{i}_{uv}}{d})} - 1}{e^{\alpha} - 1} & \text{if}\,\, D^{i}_{uv} \leq d \\ 0, & \text{if otherwise}, \end{array}\right.  \end{array} $$

where $y^{i}_{uv}$ represents the value of **y**^*i*^ at pixel (*u,v*), and $D^{i}_{uv}$ denotes the Euclidean distance between pixel (*u,v*) and its closet annotated nucleus center. *d* is a distance threshold and *α* controls the proximity value decay. With this definition, the structured label has continuous values and only a small region (controlled by *d*) around the nucleus center has positive values, as shown in Fig. 12. In this way, the model can learn to predict higher values for pixels in the central regions of nuclei.

Let *Θ* denote the FCN parameters to be learned and *Φ*(·) represent the nonlinear mapping from network inputs to outputs. Given a set of training images and corresponding proximity maps $\{\mathbf {x}^{i}, \mathbf {y}^{i}\}_{i=1}^{N}$, we estimate the parameters by minimizing the prediction error between network outputs **o**^*i*^=*Φ*(**x**^*i*^;*Θ*) and gold-standard proximity maps **y**^*i*^, *i*=1,2,...,*N*. To optimize this problem, one straightforward choice of the objective loss function is the mean squared error (MSE); however, this loss might not be suitable for our case, because a dominant portion of each proximity map has zero values and a plain MSE might lead to a trivial solution such that the predictions for all the pixels can be simply assigned zeros [67]. Thus, we adopt a weighted MSE loss that enforces model learning to pay more attention to the central regions of nuclei. Formally, the loss for the *i*-th training image is defined as

2 $$\begin{array}{@{}rcl@{}} \mathcal{L}(\mathbf{o}^{i},\mathbf{y}^{i}) = \frac{1}{2} \sum_{(u,v) \in \mathbf{y}^{i}} (y^{i}_{uv} + \lambda \bar{y}^{i}) (o^{i}_{uv} - y^{i}_{uv})^{2},  \end{array} $$

where *λ* controls the weights of the losses for different image regions. $\bar {y}^{i}$ is the mean value of **y**^*i*^ and allows the model to automatically adjust the contribution of each individual image. In practice, the $\mathcal {L}(\mathbf {o}^{i},\mathbf {y}^{i})$ is normalzied by the number of image pixels and the overall loss is the average over the entire training set.

The loss function is differentiable with respect to *Θ* and the FCN regression model is trained with the standard gradient-based backpropagation [68]. Denote **a**^*i*^ the input of the last layer for training image **x**^*i*^. The derivative of (2) with respect if **a**^*i*^ can be written as (if the sigmoid activation function is chosen in the last layer)

3 $$\begin{array}{@{}rcl@{}} \frac{\partial \mathcal{L}(\mathbf{o}^{i},\mathbf{y}^{i})}{\partial a^{i}_{uv}} = (y^{i}_{uv} + \lambda \bar{y}^{i}) (o^{i}_{uv} - y^{i}_{uv})a^{i}_{uv}(1-a^{i}_{uv}).  \end{array} $$

The derivative of the loss function with respect to the network parameters can be calculated using the chain rule for model training. During testing, the model predicts a proximity map **p** for each unseen image. Those pixels with small values, i.e., less than *ξ*· max(**p**) where *ξ*∈[0,1], are suppressed. Thereafter, nucleus centers are localized by identifying local maxima on the processed proximity map.

## Additional file

Additional file 1 Supplementary document. This supplementary document contains the precision-recall curves of nucleus detection using MiocroNet on all the 23 datasets. (PDF 153 kb)

## Data Availability

The datasets used and/or analysed during the current study are available from the corresponding author on reasonable request.

## Abbreviations

CNN: Convolutional neural network
DNN: Deep neural network
ELU: Exponential linear unit
FCN: Fully convolutional network
FN: False negative
FP: False positive
H&E: Hematoxylin and eosin
MSE: Mean squared error
NET: Neuroendocrine tumor
SVM: Support vector machine
TCGA: The cancer genome atlas
TP: True positive
