# Correction to: Towards pixel-to-pixel deep nucleus detection in microscopy images

**DOI:** 10.1186/s12859-019-3133-6

**Published:** 2019-10-22

**Authors:** Fuyong Xing, Yuanpu Xie, Xiaoshuang Shi, Pingjun Chen, Zizhao Zhang, Lin Yang

**Affiliations:** 1 Department of Biostatistics and Informatics, and the Data Science to Patient, Value initiative, University of Colorado Anschutz Medical Campus, 13001 E, 17th Pl, Aurora, CO 80045 USA; 2 J. Crayton Pruitt Family, Department of Biomedical Engineering, University of Florida, 1275 Center, Drive, Gainesville, FL 32611 USA; 3 Department of Computer and Information Science and Engineering, University of Florida, 432 Newell Drive, Gainesville, FL 32611 USA

**Correction to: BMC Bioinformatics (2019) 20:472**

**https://doi.org/10.1186/s12859-019-3037-5**

Following publication of the original article [1], we have been notified of a few errors in the html version:

- The captions for Fig. 1 and Fig. 2 have been switched
- The references to Fig. 1 and Fig. 2 have been switched within the main text

The pdf version of the article is correct. The original article has been corrected.
