# Supplementary material for: Towards pixel-to-pixel deep nucleus detection in microscopy images
Source: BMC Bioinformatics. 2019 Sep 14;20:472. doi: 10.1186/s12859-019-3037-5 (PMC6744696; doi:10.1186/s12859-019-3037-5)
Supplement: Supplementary file 1 — Supplementary document. This supplementary document contains the precision-recall curves of nucleus detection using MiocroNet on all the 23 datasets. (PDF 153 kb) [file 12859_2019_3037_MOESM1_ESM.pdf]

Supplementary Document for Towards Pixel-to-pixel Deep Nucleus Detection in Microscopy Images

Fuyong Xing, Yuanpu Xie, Xiaoshuang Shi, Pingjun Chen, Zizhao Zhang and Lin Yang

Experimental results

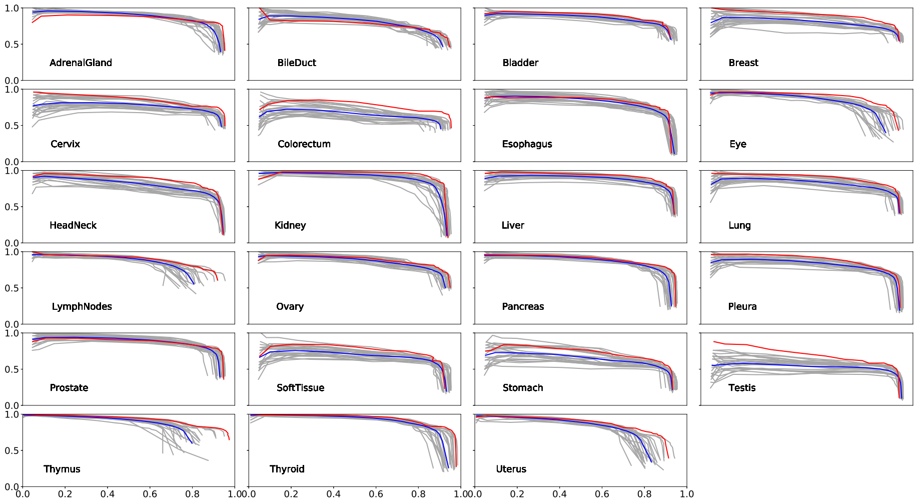

Supplementary Figure 1: Precision-recall (PR) curves of nucleus localization of MicroNet on 23 datasets. The curves are generated with varying ξ from 0 to 1. The x/y-axis represents recall/precision. For each subplot, the red curve represents the model trained and tested on the same dataset, gray curves correspond to models trained with other (or non-target) datasets and the blue curve is the average PR curve over these non-target models.
